# Low heterogeneity of tumor grades in multiple MRI-targeted prostate biopsies argues for the aggregate method of grading

**DOI:** 10.1007/s00428-025-04154-x

**Published:** 2025-06-27

**Authors:** Oliver Hommerding, Fereschte Sara Rejai, Anna Scherping, Tobias Kreft, Christine Sanders, Carsten-Henning Ohlmann, Jörg Ellinger, Phillip Krausewitz, Stefan Hauser, Manuel Ritter, Julian A. Luetkens, Marit Bernhardt, Glen Kristiansen

**Affiliations:** 1 Institute of Pathology, University Hospital Bonn, Venusberg-Campus 1, 53127 Bonn, Germany; 2 Department of Obstetrics and Prenatal Medicine, University Hospital Bonn, Bonn, Germany; 3 Department of Urology, University Hospital Bonn, Bonn, Germany; 4 Department of Urology, Johanniter-Kliniken Bonn, Bonn, Germany; 5 Department of Diagnostic and Interventional Radiology, University Hospital Bonn, Bonn, Germany

**Keywords:** Prostate cancer, MRI-targeted biopsy, Systematic biopsy, ISUP Grade Group, Aggregate method, Individual method

## Abstract

Prostate cancer diagnosis primarily relies on histological confirmation via needle core biopsy, with systematic 12-core biopsies (SB) being commonly used. Multiparametric magnetic resonance imaging (mpMRI) and MRI-targeted biopsies have shown enhanced detection of clinically significant prostate cancer. This study compares two tumor grading methods—aggregate and individual grading—used in MRI-targeted biopsies to assess their correlation with the final ISUP Grade Group (GG) of the RPE. A cohort of 108 patients with ≥ 2 positive cores in at least one MRI-targeted biopsy, totaling 179 positive lesions, was analyzed. Systematic and MRI-targeted biopsies were correlated with RPE specimens. The mean highest ISUP GG for systematic biopsies was 2.77 (SD ± 1.29), compared to 2.62 (SD ± 1.13) for targeted biopsies using the aggregate method. Comparing the highest ISUP GG in systematic as well as targeted biopsies with the final ISUP GG of the RPE, exact correlation between GG was found in 70.1% (aggregate) and 66.4% (individual) for targeted biopsies and 58.1% for systematic biopsies. The results of the individual method showed slightly better correlation with the final ISUP GG from the RPE specimen in only 0.93%, while in 2.8% of cases, it resulted in inferior correlation compared to the aggregate method. Our findings suggest that the aggregate grading method of targeted biopsies is preferable due to its comparable predictive accuracy, lower workload, and alignment with existing clinical guidelines. This supports the ISUP’s recommendation to use the aggregate method for MRI-targeted biopsies in clinical practice. Further research is needed to standardize reporting protocols for MRI-targeted biopsies and refine their integration into prostate cancer risk stratification models.

## Introduction

The diagnosis of prostate cancer is based on histological confirmation by means of a needle core biopsy. Ultrasound-guided systematic 12-core biopsy (SB) of the prostate is still the gold standard for prostate tissue sampling and the basis for the current clinical management of patients with prostate cancer. In particular, inclusion criteria for active surveillance (AS) therapy were developed based on systematic 12-core biopsy. However, upgrading rates of 20 to 30% at the time of radical prostatectomy (RPE) demonstrate the rather low sensitivity to detect significant disease using this method [1–4].

Prostate multiparametric magnetic resonance imaging (mpMRI) and MRI-targeted biopsies have been shown to increase the detection rate of clinically relevant prostate cancer due to better visualization of intraprostatic lesions, thus influencing the clinical management of patients with prostate cancer [5–13].

In contrast to systematic 12-core biopsies, in which usually one core biopsy is taken from each region, MRI-targeted biopsies often contain multiple cores from a single image-directed region or lesion. Consequently, tumor grading of targeted biopsies can be performed in two ways: the lesion can be given an overall International Society of Urological Pathology (ISUP) Grade Group (GG), which is determined by calculating the average of all cores in one biopsy or specimen container respectively (aggregate method). Alternatively, however, all cores can be graded separately (individual method).

The choice of method can lead to varying results, particularly in determining the highest ISUP GG, which can impact treatment decisions due to its crucial role in current risk assessment models. The issue was also discussed at the 2014 ISUP conference on Gleason grading of prostate cancer. Most participants report targeted biopsies by averaging the Grade of all cores from one lesion [14].

The question of whether to use the individual or aggregate method was also addressed in a few studies. A comparative study of the aggregate and individual methods found inconclusive results regarding which method correlated more closely with the final grade ISUP GG of the RPE specimen [15]. In a subsequent investigation of the same group, the aggregate method however demonstrated good performance with the well-established Memorial Sloan Kettering Cancer Center (MSKCC) pre-prostatectomy nomogram [16].

In addition to the evaluation method, other causes of heterogeneity are described. Calio et al. were able to show that the placement of the biopsy, either centrally or peripherally in the lesion, has an influence on the GG [17].

There is a notable lack of consensus among pathologists regarding the requirements for pathology reports. Further research is warranted to establish uniform criteria for reporting MRI-targeted biopsies and to integrate this novel information into risk stratification models and finally guidelines. This study aims to provide additional data on the suitability of these two grading methods, the individual and the aggregate method.

## Material and methods

All patients that underwent radical prostatectomy after prior targeted biopsy and were diagnosed at the Institute of Pathology, University Hospital Bonn, between April 2019 and June 2022 were included in the study. Material from targeted biopsy as well as RPE was available for 119 patients. Of these, 108 patients diagnosed with prostate cancer met the inclusion criteria for GG heterogeneity analysis, presenting with ≥ 2 tumor-positive cores in at least one targeted biopsy. The following data refers to the cohort of 108 patients. In total, 219 lesions were targeted and 179 (81.7%) were positive. A surgical specimen was available for all patients to correlate the data obtained from the biopsy material to the final tumor grade, referring to the global Gleason score assigned to the (RPE) specimen. The median age was 65 (51 to 82) and the initial PSA mean ± SD was 11.0 ± 12.0 ng/mL. Additional clinico-pathological data are summarized in Table 1. Analysis and tumor grading was performed by a board-certified pathologist (OH) using classical glass slides. For each target, the number of cores obtained and the number of positive cores were recorded. In addition to the individual grade, tumor length and growth fashion (continuous/discontinuous) were noted.

**Table 1:** Clinicopathological data

Clinical features	Finding (*n* = 108)
Age, median (range), years	65 (51–82)
Initial PSA, ng/ml, mean (range, SD)	11.0 (0.95–88, 12.0)
Pathological parameters	
RPE parameters	
Grade group	
1 (Gleason score 3 + 3 = 6), *n* (%)	4 (3.7%)
2 (Gleason score 3 + 4 = 7a), *n* (%)	61 (56.5%)
3 (Gleason score 4 + 3 = 7b), *n* (%)	26 (24.1%)
4 (Gleason score 4 + 4 = 8, 3 + 5 = 8, 5 + 3 = 8), *n* (%)	3 (2.8%)
5 (Gleason score 4 + 5 = 9, 5 + 4 = 9), *n* (%)	13 (12.0%)
Neoadjuvant therapy	1 (0.9%)
Organ-confined disease, *n* (%)	62 (57.4%)
Extraprostatic extension, *n* (%)	30 (27.8%)
Seminal vesicle involvment, *n* (%)	16 (14.8%)
Infiltration of bladder, pelvic wall, rectum	0
Lymph node positive, *n* (%)	10 (9.3%)
Positive margins, *n* (%)	23 (21.2%)

Data analysis was performed using Microsoft Excel (Microsoft Office Professional Plus 2026, Redmond, Washington, USA) and IBM SPSS Statistics (IBM SPSS Statistics Version 29.0.2.0, IBM, New York, USA). For group comparisons of metric data, the Mann–Whitney *U* test and the Kruskal–Wallis test were performed. For nominal scaled data, the McNemar test was used for analysis. A *p*-value of < 0.05 was considered statistically significant.

## Results

A total of 108 patients who received a systematic and targeted prostate biopsy and showed ≥ 2 positive cores in at least one targeted biopsy (TB) and thus met the inclusion criteria for the analysis of GG heterogeneity were included in the study. Regions suspicious for cancer (region of interest (ROI)) were represented by cores in a single specimen container. No ROI was divided over several specimen containers. The median age was 65 (51 to 82) years and the initial PSA mean ± SD was 11.0 ± 12.0 ng/µL.

A total of 219 lesions were biopsied of which 179 lesions were positive (81.7%). The mean highest ISUP GG in systematic biopsies was 2.77 (SD ± 1.29, median 2) compared to 2.62 (SD ± 1.13, median 2) in targeted biopsies, which were initially analyzed applying the aggregate method.

**Fig. 1:**
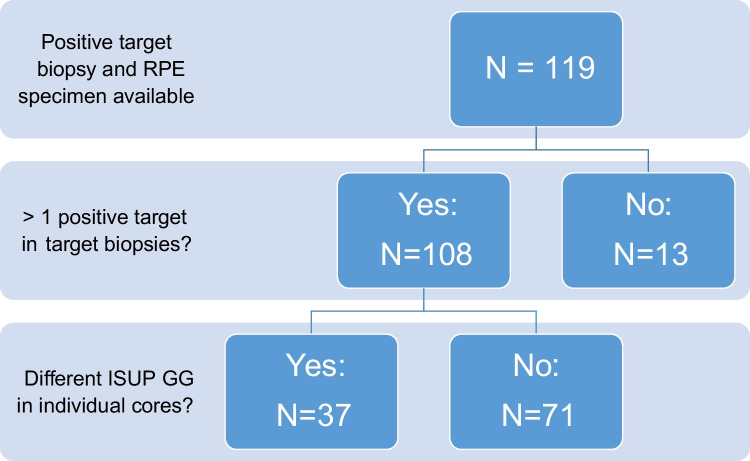
Flow chart of the case selection process

**Fig. 2:**
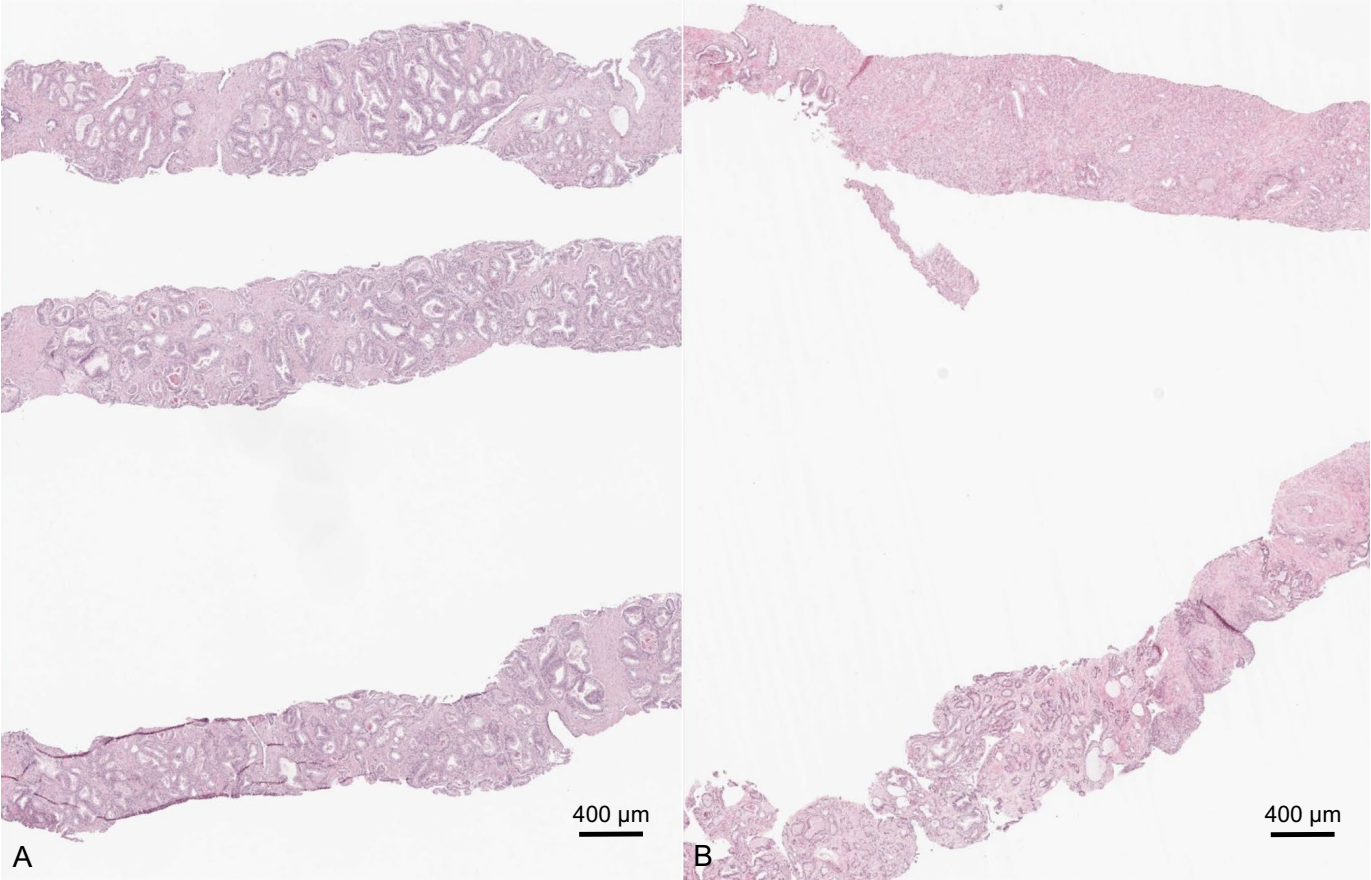
Exemplary images of target biopsies with similar GG (**A**) and differing GG (**B**) between cores

To investigate whether the individual grading of single cores in TB enables a more accurate prediction of the final ISUP GG, individual grading of all targeted biopsies was performed. Of note, the majority of patients had an identical ISUP GG in the individual cores of the targeted biopsies (71/108 patients, 65.7%). Only 37 patients (34.3%) showed individual cores with differing ISUP GG when applying the individual grading method (Fig. 1). Of these, several patients showed multiple targeted lesions with differing GG of the single cores, so that a total of 46 targeted lesions of 37 patients could be included in the analysis (Fig. 2). The difference between ISUP GG of the individual cores was between 1 and 3 (mean 1.23 ± 0.52, median 1). In most of these patients (31/37, 83.8%), the differing ISUP GG in the individual cores did not lead to an upgrade of the lesion. There was no significant difference in the percentage of positive cores between lesions that did or did not show a difference in final ISUP GG (*p* = 0.73).

In 14 patients (13.0%), tumor was identified in the targeted biopsies only. In the remainder of 94 cases, tumor was present in both systematic and targeted biopsies. The mean percentage of positive cores was 34.5% for SB and 84.53% for TB. Out of those patients, in which both systematic and TB contained cancer, the highest ISUP GG of systematic biopsy and the highest ISUP GG of the targeted biopsy were compared. When using the individual method to compare SB and TB, the highest GG was the same in 64.9% (*n* = 61). When comparing highest GG of SB and TB using the aggregate method, the highest GG was the same in 63.8% (*n* = 60). ISUP GG was higher in SB in 14.9% (*n* = 14) or higher in TB in 20.2% (*n* = 19) in individual grading and higher in SB in 20.2% (*n* = 19) or higher in TB in 16.0% (*n* = 15) in aggregate grading. In only 9 lesions (5.0% of all 179 positive lesions), an upgrading was observed when applying the individual method, mostly from ISUP GG 2 to 3 (6 lesions, 3.4%).

To assess the performance of the aggregate method of grading targeted biopsies, the highest ISUP GG of the targeted biopsies was correlated with the RPE specimen. In RPE specimens, final grade group was assessed as the global grade. One patient had received androgen deprivation therapy so that GS was not applicable and was therefore excluded from further analysis. Exact correlation of ISUP GG of SB and RPE was found in 58.1% (*n* = 54 out of *n* = 93 with positive SB) patients. The percentage of positive cores did not show an impact on the results (*p* = 0.978).

Out of the 37 patients with divergent GG when comparing individual and aggregate method, the highest GG was found in a different target in 10 patients. For the remainder of 26 patients, exact correlation of ISUP GG of TB and RPE was found in 16 patients for individual and 20 patients for aggregate method leading to an overall correlation on 66.4% (*n* = 71) for individual and 70.1% (*n* = 75) for aggregate grading methods. The difference between the two methods was not statistically significant (*p* = 0.125). Additionally, the percentage of positive cores had no impact on the result (*p* = 0.386 individual and *p* = 0.600 aggregate method). Both targeted and systematic biopsies correlated with the final ISUP GG of the RPE specimen in 41.1% (*n* = 44) for individual and 43.0% (*n* = 46) for the aggregate method.

The correlation of these newly collected data with the final GG of the RPE specimen revealed that grading of targeted biopsies according to the individual method led to a better correlation in 1 patient (0.93%), while in 3 patients (2.8%) the now upgraded lesion showed an inferior correlation in comparison to the aggregate method.

## Discussion

In the dataset presented here, two different grading methods of targeted prostatic biopsy cores were performed in order to understand if grading each core separately - in contrast to assigning an overall Gleason Score and ISUP GG on all cores present in one specimen container - would lead to divergent results. In the majority of cases, the different grading methods did not lead to a difference in GG of the respective lesion nor an upgrade of the whole case.

Targeted prostatic biopsies following mpMRI of the prostate have become a valuable tool improving sensitivity and detection rates of clinically significant prostate cancer [10]. To date, specifically targeting lesions being highly suspicious for prostate cancer according to PI-RADS (Prostate Imaging-Reporting and Data System) Scores 3–5 is a complementary method to the classical and long established method of systematic biopsy which aims at a representative presentation of the organ [18]. Treatment modalities and therapeutic decisions such as eligibility for active surveillance are still dependent on the histopathological result of conventional 12-core systematic biopsy, although subsequent MRI with confirmatory biopsy within 6 months in men who did not receive pre-biopsy MRI is recommended [18, 19]. However, as mpMRI should be offered to all or at least the majority of patients according to several national guidelines, the importance of targeted biopsies on subsequent treatment decisions is most likely going to increase in close future [20, 21]. In contrast to systematic biopsies, where each region is represented by a single core and often sent for histopathological evaluation in individual specimen containers, targeted biopsies mostly consist of several biopsies, which are often sent in the same container [22]. Also in contrast to systematic biopsies, where guidelines recommend to assign an individual grade per core/geographic region—the so-called individual method—the current recommendation, following the 2019 consensus conference of the International Society of Urological Pathology (ISUP) as well as a similar conference by the Genitourinary Pathology Society (GUPS), is to assign an overall grade on all cores present in a single specimen container, originating from the same lesion [20, 23, 24]. This is called the *aggregate* (*or global*) *method*. The only exception to the rule may be, if cores were inked by urologists prior to histopathological examination [23, 25]. Given that targeted biopsies aim to represent a single tumor focus defined by radiology, an aggregate approach seems legitimate. From a practical point of view, several advantages may arise from the aggregate method. First of all, assigning an overall grade is time efficient. This truly is of importance in a setting of pathologist shortage and high workload [26]. Secondly, prostatic biopsy tissue is fragile and fragmentation is an issue. Given that handling of tissue becomes increasingly difficult with the amount of cores per container, one might end up with numerous fragments on the slide although the number of cores taken, as indicated on the request form, is significantly lower. This is becoming even more of an issue with a higher number of cores [27]. A potential, though extremely important risk arising from fragmentation may be overscoring. This may be true in cases where smaller fragments, that, due to tumor heterogeneity, only contain high grade parts of the tumor, that otherwise is present as a mixture of high and low grade areas, are scored according to the individual method [28]. Also, as high grade areas with geographic cribriform or solid epithelial proliferations are more prone to fragmentation, this is an important consideration.

In our attempts to improve patient care, convenience for the pathologist is of subordinate importance. Differences in ISUP GG in a core biopsy series may influence length of subsequent androgen deprivation therapy (ADT) following definitive radiation therapy [20]. Using the grading method that best reflects patient prognosis and final GG at RPE therefore is crucial. Numerous studies have demonstrated a correlation between stage and/or prognosis and number of positive cores, linear tumor extent, and Gleason Score for systematic biopsies [29–31]. In targeted biopsies, these findings cannot be applied alike. This is also due to the fact that there is no general standard for how many cores should be taken from a nodule that appears suspicious on imaging. However, a minimum of three to five cores per lesion is recommended [18]. Many risk calculators and treatment algorithms rely on the highest GS or GG in a biopsy series. Therefore, it is crucial that reported grade most accurately reflects the definitive grade of the tumor as determined on RPE. The good news here is that in the majority of cases, GG will not differ whether grading is performed according to individual or aggregate method. In the present cohort, no difference between GG of individual cores was observed in 65.7% of lesions. If GG differed, highest GG of the lesion remained unchanged in more than 90% of patients as well as targeted lesions investigated. This was in concordance with previously published data by Gordetsky et al., who investigated a cohort of 55 patients and found a change of GG between individual and aggregate method for only three patients [15]. In a similar setup, Deng et al. analyzed a large cohort of 317 patients and found a concordance of at least 72%. However, the authors did include the GG of the core with the longest/largest cancer as an additional factor in their analysis, potentially decreasing overall agreement [32]. More pronounced heterogeneity was reported by Mesko et al., who described a difference in GS in 55% of targets. A limitation to their study remains the small number of cases (*n* = 51) and the lack of analysis of GG. In conclusion, heterogeneity may be of importance in some, though rather a minority of cases so that the choice of grading method mostly leads to no difference regarding patient care.

Gleason score in RPE is the most important predictor of biochemical recurrence [33]. Accurately predicting RPE GS is crucial, not only for those patients opting for surgery but also those treated by definitive radiotherapy without a RPE specimen available to correct diagnosis. For systematic biopsies, an upgrade of GS in 20–30% at RPE has been described in the literature [34]. Targeted biopsies are significantly more likely to detect clinical significant cancer, so that one might assume a better concordance with RPE GG, given that the dominant cancer has been sampled with a higher likelihood as compared to SB [10]. In our cohort, GG concordance was higher for targeted biopsies than for systematic biopsies (70.1% aggregate and 66.4% individual vs. 58.1% systematic), matching previously published data which describes a range of 29.3–68.4% for the concordance of individual and 47.2–72.3% for the concordance of aggregate method grading and final GG of subsequent RPE [32, 35, 36]. On a per case level, the individual method resulted in better alignment for 1 patient (0.9%), while 3 patients (2.8%) demonstrated poorer correlation compared to the aggregate method. This indicates that while the individual method may provide a slight advantage in certain cases, it does not consistently outperform the aggregate method.

In summary, our data endorses that the aggregate method for grading targeted biopsies is slightly superior to the individual method. Furthermore, the individual method is associated with an increased workload and does not offer consistent advantages in clinical correlation with final pathology outcomes. Based on these findings, we recommend the use of the aggregate method, a stance supported by other studies and aligned with the ISUP and GUPS guidelines [14–16, 24]. By adopting the aggregate method for targeted biopsies, pathologists can streamline biopsy interpretation while maintaining accuracy and clinical relevance in prostate cancer management.

## Data Availability

Data can be obtained from the corresponding author upon reasonable request.
